# Childhood-Onset Takayasu Arteritis: Clinical Features of Disease and Relapse Risk Factors

**DOI:** 10.3390/children12010070

**Published:** 2025-01-08

**Authors:** Vera Podzolkova, Galina Lyskina, Olga Shpitonkova, Angelina Polyanskaya, Svetlana Chebysheva, Marina Shakhnazarova, Jinbo Zhao, Aleksandr Suvorov, Vera Khudoroshkova, Natalia Geppe

**Affiliations:** 1 Department of Children’s Diseases, N.F. Filatov Clinical Institute of Children’s Health, I.M. Sechenov First Moscow State Medical University, 119435 Moscow, Russiameleshkina_a_v@staff.sechenov.ru (A.P.); chebysheva_s_n@staff.sechenov.ru (S.C.); shakhnazarova_m_d@staff.sechenov.ru (M.S.); geppe_n_a@staff.sechenov.ru (N.G.); 2 Department of Internal, Occupational Diseases and Rheumatology, N.V. Sklifosovsky Institute of Clinical Medicine, I.M. Sechenov First Moscow State Medical University, 119435 Moscow, Russia; chzhao_ts@student.sechenov.ru; 3 World-Class Research Center “Digital Biodesign and Personalized Healthcare”, I.M. Sechenov First Moscow State Medical University, 119048 Moscow, Russia; suvorov_a_yu_1@staff.sechenov.ru; 4 N.V. Sklifosovsky Institute of Clinical Medicine, I.M. Sechenov First Moscow State Medical University, 119048 Moscow, Russia; khudoroshkova_v_d@student.sechenov.ru

**Keywords:** Takayasu’s arteritis, systemic vasculitis, ITAS.A score

## Abstract

**Background:** Takayasu’s arteritis (TA) is a systemic vasculitis that primarily affects the aorta and major arteries. Despite aggressive treatment with glucocorticoids (GCs) and non-biological disease-modifying antirheumatic drugs (nbDMARDs), about 30% of patients experience resistance to therapy or relapse. This study aimed to identify risk factors associated with refractory and relapse TA in pediatric patients. **Methods:** A retrospective, open-label, case–control study was conducted with 56 pediatric patients with TA diagnosed between February 2011 and October 2022. Fourteen patients were excluded due to insufficient data in their medical records, leaving 42 for further analysis. The patients were divided into two groups: Group 1 (18 patients) with no evidence of relapse and Group 2 (24 patients) with relapse despite first-line treatment at the end of the follow-up period. Clinical, laboratory, and instrumental data were collected and analyzed using R v4.2 and Python v3.10. **Results:** The median time to relapse was 18 [IQR: 13; -] months according to the Kaplan–Meier curve. Patients with ITAS.A with a diagnosis of TA ≥ 12 had a higher probability of relapse, according to the log-rank criterion (*p* = 0.006). Symptoms of critical ischemia, such as limb claudication, were more common in Group 2 at diagnosis (*p* = 0.047), and a trend toward a longer diagnostic delay was observed (*p* = 0.067). **Conclusions:** Pediatric patients with an initial ITAS.A score above 12 have a higher risk of relapse when treated with a combination of GCs and nbDMARDs as first-line treatment. Further research is needed to identify high-risk patients more accurately and optimize therapeutic strategies.

## 1. Introduction

Takayasu’s arteritis (TA) is a rare form of systemic vasculitis that primarily affects large blood vessels, particularly the aorta and its main branches [1]. It is the third most prevalent pediatric vasculitis after IgA-related vasculitis and Kawasaki disease [2]. The exact causes of TA are unknown. Still, it is believed to be linked to abnormal immune responses in the arterial walls, leading to endothelial cell proliferation, neovascularization, fibrosis, and damage to elastic fibers [3,4].

According to recent guidelines from the European League Against Rheumatism (EULAR) [5] and the American College of Rheumatology (ACR) [6], the first-line treatment for TA typically involves a combination of glucocorticoids (GCs) and non-biologic disease-modifying medications (nbDMARDs), such as methotrexate (MTX) and cyclophosphamide (CYC). However, according to various studies, the response rates to standard treatment are often below 60–70% [7,8,9]. Focusing on adult patients with TA, Goet et al. [7] found that 5-year relapse-free survival was around 66%, but it dropped to 52% after a decade. Uncovering potential indicators of refractory or relapsed TA and finding ways to prevent these outcomes is of great academic interest, as it could improve the prognosis of the disease. This study aims to identify risk factors that correlate with refractory and relapse manifestations of TA in children under observation.

## 2. Materials and Methods

A retrospective clinical study was conducted from 2021 to 2023 at the Clinic of Children’s Diseases, Sechenov’s Center of Maternity and Childhood. The study included 56 children who met the study criteria (Table 1). The optimal sample size was not calculated because the disease is rare, and all available patients were recruited.

The study protocol was approved by the Interuniversity Ethics Committee of I.M. Sechenov First Moscow State Medical University (protocol No. 06-21, dated 7 April 2021). Informed consent was obtained from all participants. For children under the age of 15, consent was provided by a parent or legal representative, while patients aged 15 years or older provided their own consent.

In the initial phase of this study, patients’ enrollment took place during their inpatient care at Pediatric Rheumatology Departments No. 1 and No. 2 of Sechenov’s Center of Maternity and Childhood, the Clinic of Children’s Diseases, between February 2011 and October 2022. Inclusion was based on archival medical records confirming a TA diagnosis, established either after 2011 or retrospectively verified before 2011, in accordance with the criteria of the European League Against Rheumatism (EULAR), the International Organization for Research in Pediatric Rheumatology (PRINTO), and the Pediatric Rheumatology European Society (PRES) starting from 2010 [10].

The overall male-to-female ratio in this study was approximately 1:5.2, with no significant sex differences observed among patients experiencing the onset of TA at or before the age of 7. Among the nine patients with onset before the age of 7, four were male (M/F ratio 1:1.25), whereas the M/F ratio among those with onset after the age of 7 was 1:8.4. The median age of onset was 11.5 (IQR: 9.5; 14) years.

The delay in diagnosis ranged from 1 to 120 months, with a median duration of 10 (IQR: 3; 22.5) months. Two mothers of patients (3.57%) were also diagnosed with TA, and one child (1.79%) had a family history of psoriasis. The remaining 94.2% of patients had no hereditary predisposition to rheumatic diseases.

**Figure 1:**
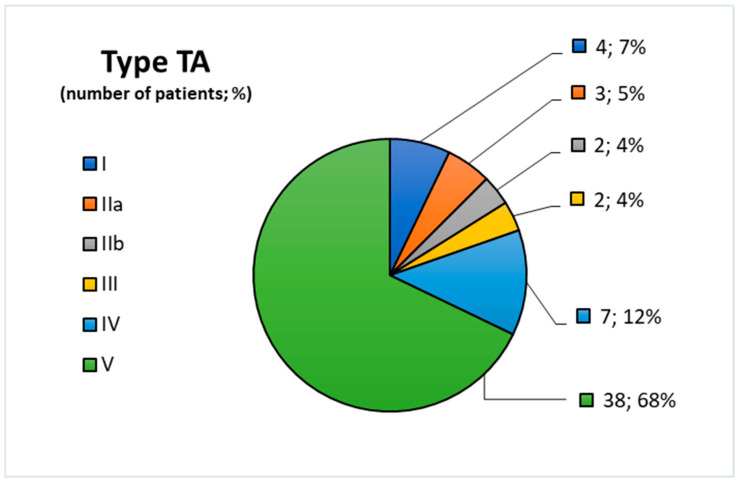
Distribution of patients by type of TA (n = 56): type I—7% (n = 4); type IIa—5% (n = 3); type IIb—4% (n = 2); type III—4% (n = 2); type IV—12% (n = 7); type V—68% (n = 38).

The type of TA was determined by our team based on the angiographic classification by Hata et al. [11]: type I primarily involves the branches from the aortic arch; type IIa involves the ascending aorta, aortic arch, and its branches; type lIb involves the ascending aorta, aortic arch with its branches, and thoracic descending aorta; type III involves the thoracic descending aorta, abdominal aorta, and/or renal arteries; type IV affects only the abdominal aorta and/or renal arteries; and type V affects the combined features of both type lIb and IV. According to our findings, the most common form of vascular involvement was type 5 TA, observed in 38 patients (68%). The second most common was type 4, affecting seven patients (12%). Type 1 was noted in 4 patients (7%), type 2a in 3 patients (5%), and types 2b and 3 in 2 patients each (4%). The distribution of patients by TA subtype is presented in Figure 1.

Clinical and laboratory data analysis and an evaluation of the disease course and outcomes were conducted for 45 patients because 11 patients lacked sufficient data in their medical records for this study. All these 45 patients underwent regular (each 6–8 months) laboratory and imaging studies, with ultrasound being the primary method used in all cases. To confirm the diagnosis, 35 children underwent computed tomography (CT), and 10 underwent catheter angiography. To assess the progression of vascular injury under treatment, an ultrasound examination of the vessels was performed at each hospitalization, and if necessary, CT angiography, MR angiography, or PET-CT were conducted. For laboratory studies, we performed a blood count and considered the erythrocyte sedimentation rate (ESR), C-reactive protein (CRP), and biochemical and immunological blood parameters. The Indian Takayasu Activity Score with acute-phase reactants (ITAS.A [12]) was elevated in all 45 patients at the time of diagnosis confirmation, with a median value of 13 (IQR: 10; 15.5).

Following the confirmation of a TA diagnosis, 42 patients were initiated with a combination of GCs and nbDMARDs, with a median starting GC dose equivalent to 1 (IQR: 0.61; 1) mg/kg/day of prednisolone. Among the nbDMARDs, MTX was the most commonly administered, prescribed to 38 patients at a median dose of 12 (10 to 12.5) mg/m^2^/week. CYC was used in seven patients at a dose of 500 mg/m^2^/month. IV Tocilizumab was added to the combination therapy for three patients at a rate of 8 mg/kg per month as the first-line treatment. These patients were not included in the relapse predictor study due to the limited number of cases (n = 3).

At the time of diagnosis, 16 patients (35.6%) underwent intravenous pulse therapy with methylprednisolone due to high inflammatory activity. A favorable response to first-line treatment within the first 12 months was observed in 29 out of 45 patients, marked by a reduction in inflammatory activity and the absence of TA progression, as confirmed by imaging and other research methods. Six patients were monitored for less than 12 months; however, during their last clinic visit, these patients also displayed no signs of TA activity.

To identify the risk factors for TA relapse, we divided the 42 patients into two groups based on the presence or absence of relapse at the end of the follow-up period: Group 1 (n = 18) included patients who did not show signs of disease activity during treatment with first-line non-biologic DMARDs, and Group 2 (n = 24) comprised patients with signs of disease activity during this treatment. A retrospective open comparative case–control study was conducted between these groups to identify potential predictors of TA relapse in children during primary treatment.

Statistical analysis was performed using R v4.2 and Python v3.10. For quantitative variables, the distribution type was assessed using the Shapiro–Wilk test, and measures such as the median, interquartile range (Q1–Q3), and minimum and maximum values were calculated. For categorical and qualitative characteristics, proportions and absolute counts were determined. The comparative analysis employed the Welch *t*-test (for two groups) for normally distributed quantitative characteristics and the Mann–Whitney U test (for two groups) for non-normally distributed quantitative characteristics. Comparative analysis of categorical and qualitative characteristics was conducted using the Chi-square–Pearson test, and when it was inapplicable, Fisher’s exact test was used. The level of statistical significance was set at 0.05. Fisher’s exact test was used where sample sizes were small.

Univariate regression analysis was conducted using the Cox regression to assess the time to the first relapse and the influencing factors. Kaplan–Meier curves and life tables were employed to estimate the time to the first relapse. In cases where it was not possible to calculate the interquartile range or average survival time due to a small number of patients achieving the desired outcome, a dash was used to indicate incomplete or insufficient data.

The ITAS.A index was a primary variable in this study. Through ROC analysis and the calculation of the highest Youden index, a threshold value for this indicator concerning relapse risk was determined.

An artificial intelligence algorithm using a random forest model was utilized to identify factors influencing the risk of a first relapse. Given the limited sample size, simple regression analysis would have been underpowered; hence, the feature selection strategy focused on maximizing variance, reducing bias, and constructing a robust model to identify potential predictors of relapse. No separation of training and test datasets was performed, but extensive cross-validation was applied.

The predictor selection pipeline consisted of 500 iterations. Each iteration involved a random selection of 25% of the entire dataset, followed by data transformation. For quantitative data, missing values were imputed using Bayesian ridge regression, and quantile transformation was applied to achieve a normal distribution. For categorical data, the most frequent category was imputed for missing values, followed by one-hot encoding. The random forest classifier, using 800 estimators, was then fitted to determine feature importance. The median feature importance for each factor was calculated over the 500 iterations.

To assess the relationship between features and outcomes, the 5, 10, and 15 most significant features were selected. Three models were subsequently rebuilt using the same data transformations, with a random forest consisting of 1000 estimators employed as the classifier. Cross-validation with four folds and data shuffling was performed to optimize hyperparameters. The results were evaluated using the time-dependent area under the ROC curve (AUC) and the concordance index.

Mathematical modeling was performed using Python version 3.11 and the scikit-survival library [13].

## 3. Results

### 3.1. Demographic and Clinical Features

When comparing the groups, no significant differences were observed between Group 1 (patients without relapse with first-line therapy with nbDMARDs) and Group 2 (patients with relapse with first-line therapy with nbDMARDs) in terms of sex distribution (*p* > 0.999) or age at the onset of TA (*p* = 0.504). However, it is noteworthy that Group 2 exhibited higher median values for the overall disease course (*p* = 0.040) and the length of the follow-up period in our clinic (*p* < 0.001). In addition, the duration of TA before diagnosis was slightly longer for Group 2 patients, although this difference was not statistically significant (*p* = 0.067). This suggests that a longer delay in diagnosis may be associated with an increased risk of the recurrence of the condition. A comprehensive summary of the demographic data for both Group 1 and Group 2 is provided in Table 2.

Within Group 2, only four children (16.7%) received a TA diagnosis within the first six months from symptom onset, compared to eight children (44.4%) in Group 1. Within 12 months from symptom onset, the diagnosis was established in 61.1% of patients in Group 1 and 50% in Group 2. It is noteworthy that the duration of observation in Group 1 was statistically significantly shorter (*p* = 0.001) than that in Group 2, suggesting that some Group 1 patients may not have been observed long enough for a TA relapse to occur.

As the first symptoms of TA, 40 out of 45 patients reported general malaise at the onset of the disease. Fever, headaches, and vascular pain were also commonly observed. At the onset of TA, limb claudication and a weakened or absent pulse were noted in about 25% of patients. Less common symptoms accompanying the onset of the condition included hearing impairment, reduced visual acuity, loss of consciousness, and dizziness.

When comparing the symptoms at the onset of TA in both groups, malaise was the most frequently observed (Group 1—91.1%; Group 2—87.5%), followed by fever and headache. Among patients in Group 2, complaints of limb claudication were significantly more prevalent (*p* = 0.047), while abdominal pain was common but did not reach statistical significance (*p* = 0.109), which indicates critical ischemia of target organs. A detailed breakdown of clinical manifestations and their respective frequencies is provided in Table 3.

Twelve patients were diagnosed with arterial hypertension, marked by an elevation above the 95th percentile for height. Bruits were detected in 28 patients (62.2%) during auscultation. Of the hypertensive children, 13 required surgical intervention due to the primary condition, with seven undergoing surgery within the first six months following diagnosis. In efforts to discern factors contributing to the onset of critical artery stenosis necessitating surgical intervention, the only statistically significant finding was that patients who underwent surgery had experienced a more prolonged period prior to diagnosis (*p* = 0.013).

At diagnosis, the median ESR stood at 49 (IQR: 38; 61) mm/h, and the median CRP level was gauged at 38 (IQR: 15; 92.9) mg/L. Additionally, 80% of the patients displayed symptoms of anemia and leukocytosis, while 42.5% exhibited thrombocytosis. Of the 45 patients, 23 (51.1%) were administered antibacterial therapies, with up to five courses per child, as their initial presentations were misinterpreted as infection.

### 3.2. Vascular Involvement

As shown in Table 4, the most commonly affected arteries were the left carotid artery (66.7%), abdominal aorta (51.1%), and left subclavian artery (51.1%). The least affected arteries included the splenic, femoral, iliac, and right vertebral arteries. Notably, no involvement of the pulmonary or coronary arteries was observed at the time of diagnosis. The involvements of the descending thoracic aorta, abdominal aorta, and superior mesenteric artery were slightly more common in patients from Group 2.

**Figure 2:**
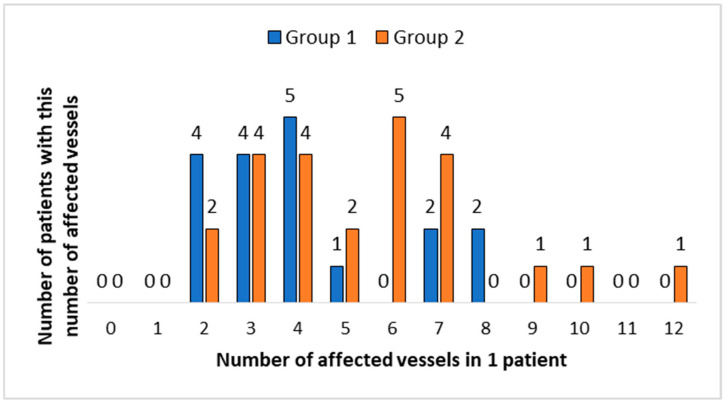
Distribution of patients in Group 1 and Group 2 depending on number of affected vessels.

The medians of the number of vessels involved in the pathological process did not significantly differ between the two groups, amounting to 4 (IQR: 3; 4.75) for Group 1 and 5.5 (IQR: 3.75; 7) for Group 2. However, there was a tendency for the number of affected vessels per patient to be higher in Group 2 than in Group 1 (Figure 2).

### 3.3. Relapse and Its Risk Factors

The relapse of TA in our cohort was most commonly identified through the progression of vascular lesions (91.7%) and the emergence of laboratory markers of inflammation (83.3%). The median CRP level at relapse was 9.45 mg/L (interquartile range: 6 to 18 mg/L), and the median ESR was 29.5 mm/h (interquartile range: 20.5 to 43.5 mm/h). The most frequently reported clinical symptoms during relapse included malaise (70.8%), headache (54.2%), and pain along the affected vessels (33.3%). The ITAS.A score was elevated in all Group 2 patients at the time of relapse, with a median value of 7 (interquartile range: 4.5 to 9.5).

Overall, patients with refractory and relapse TA experienced a statistically significant delay in obtaining an accurate diagnosis and initiating appropriate therapy. At the time of diagnosis, Group 2 patients were more likely to present with symptoms indicative of critical ischemia, such as abdominal pain and intermittent limb weakness.

**Figure 3:**
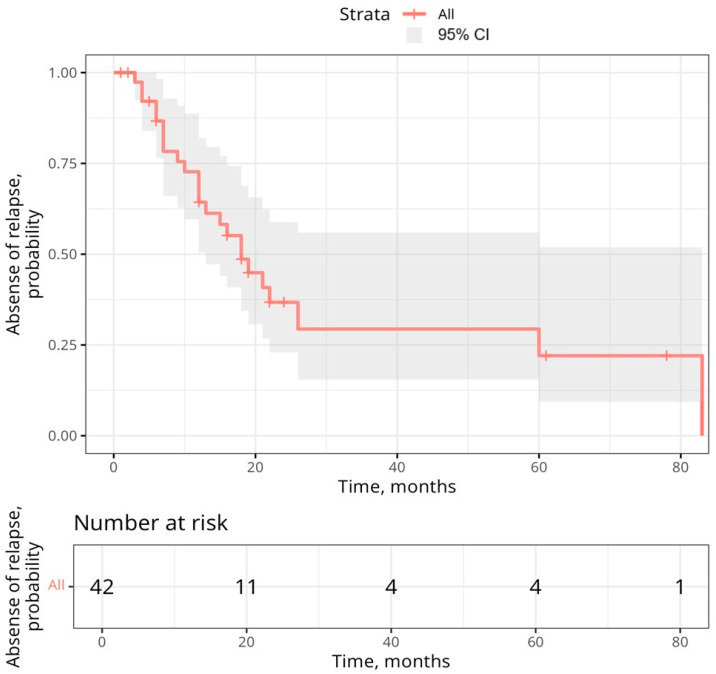
The Kaplan–Meier curve for the development of relapse. Red line—the median time to relapse, the shaded area—95% CI.

The Kaplan–Meier curve for the development of relapse is presented in Figure 3.

The Kaplan–Meier curve shows an approximately 50% chance of survival without signs of relapse until the end of the 18-month follow-up period among all patients with first-line therapy. The median time to relapse was 18 [13; -] months.

We investigated the threshold value of the ITAS.A at the time of diagnosis, which suggested a higher probability of relapse in our patients. Using ROC analysis according to Youden’s method, the threshold for the ITAS.A was determined as 12. For this threshold, the AUC was 0.701 [0.61; 0.728], the Se was 0.791 [0.676; 0.898], the Sp was 0.611 [0.465; 0.665], the PPV was 0.731 [0.578; 0.832], and the NPV was 0.687 [0.539; 0.828]. Thus, this threshold was more likely to confirm rather than exclude the probability of relapse according to a higher sensitivity and positive predictive value, but as a single predictor, it showed a relatively moderate association (Table 5).

**Figure 4:**
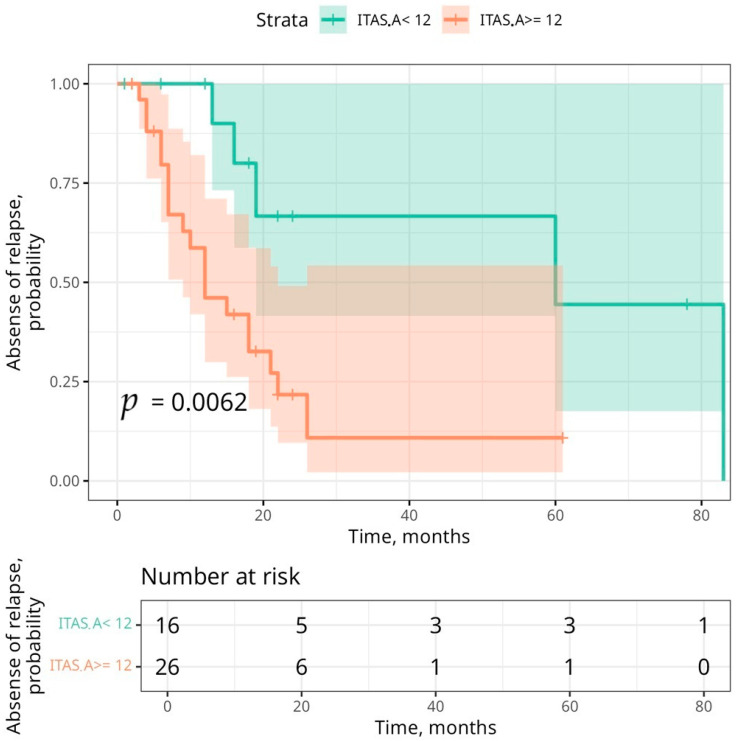
Time to relapse in patients due to ITAS.A score.

Thus, in the group with a lower ITAS.A score, relapse occurred after a more extended period; the significance of the log-rank criterion was *p* = 0.006 (Figure 4).

After building 500 random forest models and averaging the significance of predictors from the models, a table of features with median importance was generated (in descending order of significance) (Table 6).

**Figure 5:**
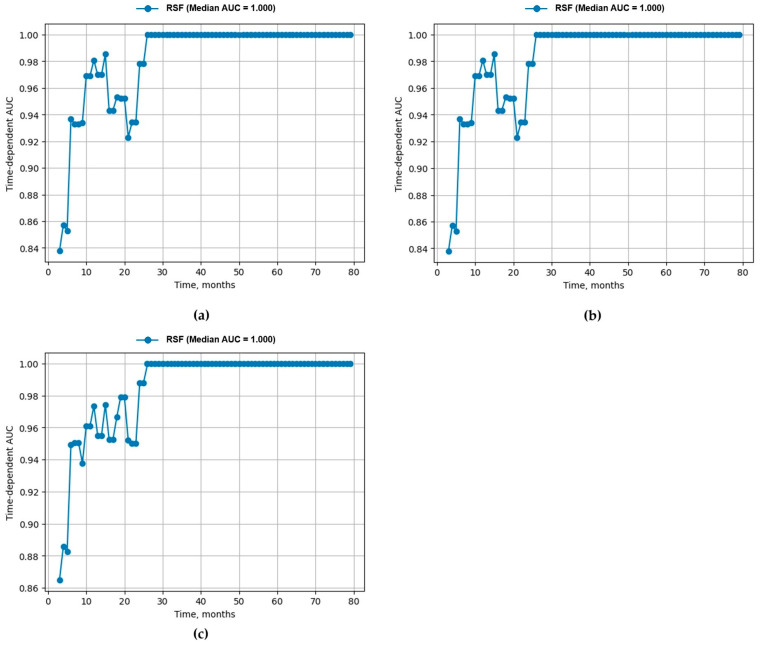
The time-dependent ROC-AUC for the following. (**a**) Five predictors: ITAS.A at diagnosis, PLT at diagnosis, time to diagnosis, GC dose at diagnosis, and age at onset. (**b**) Ten predictors: ITAS.A at diagnosis, PLT at diagnosis, time to diagnosis, GC dose at diagnosis, age at onset, WBC at diagnosis, fever at onset, bruits at diagnosis, CRP at diagnosis, and arterial hypertension at diagnosis. (**c**) Fifteen predictors: ITAS.A at diagnosis, PLT at diagnosis, time to diagnosis, GC dose at diagnosis, age at onset, WBC at diagnosis, fever at onset, bruits at diagnosis, CRP at diagnosis, arterial hypertension at diagnosis, ESR at diagnosis, HGB at diagnosis, asymmetry of BP at diagnosis, abdominal pain at onset, and vascular pain at onset.

The time-dependent ROC-AUC is significant and shows stable estimates over the entire period for each number of predictors (Figure 5a–c).

When retraining the model with the first five, ten, and fifteen factors from the table, a fairly high connection was observed between the predictors and the outcome. In Table 6, we see the value of the concordance index and average ROC-AUC depending on the number of predictors in the models (Table 7).

In all three models, the AUC improved in 10 and more months, and we can observe that a compact model with ten predictors was as good as a model with fifteen predictors.

## 4. Discussion

Managing patients with TA is inherently complex, primarily due to diagnostic delays and low responsiveness to first-line therapy [14]. The challenges in diagnosing TA in pediatric populations are partly attributable to a general lack of awareness among primary care physicians and the unique progression of the disease within these age groups [15,16].

Our study emphasizes the lack of significant sex differences in the incidence of TA among children under seven, which is consistent with prior studies [17,18]. Additionally, we observed that 51.1% of our study cohort exhibited the involvement of the abdominal aorta in the pathological process. Notably, the fourth type of TA, as defined by angiographic classification (i.e., the isolated involvement of the abdominal aorta and its branches), emerged as the second most prevalent type in our cohort, representing 12% of cases. These observations align with the findings of other studies on TA patients [17,19,20,21,22].

At the point of diagnosis, the most frequently observed ischemic symptoms of TA included pulse weakening or absence (33.3%), hypertension (26.7%), and abdominal pain (20%). Initial manifestations of TA predominantly comprised symptoms indicative of systemic inflammation, such as malaise (91.1%), fever (68.9%), and headaches (53.3%). These findings contrast with Watanabe et al.’s [23] study on adults, where these symptoms were present at rates of 12.1%, 34.7%, and 8.2%, respectively. Moreover, all patients demonstrated elevated ESR and CRP levels. These findings, along with nonspecific systemic inflammation symptoms, often led primary care physicians to misinterpret the condition as an infectious disease. Consequently, half of the children with TA in our study received between one and five courses of antibiotics before an accurate TA diagnosis was established.

Kasai et al.’s [24] research suggests that 5% of pediatric patients with unexplained fevers ultimately receive a TA diagnosis. Similarly, Nozawa et al. [25] have shown that ultrasound doppler scanning (UDS) and multi-slice computed tomography (MSCT) have comparable diagnostic efficacies for TA in children with unexplained fevers. Hassold et al. [26] suggest that pediatric patients with TA may require more careful monitoring and the more frequent use of biological medications compared to adults, due to the severity of the disease. Therefore, utilizing UDS as a preliminary screening tool for early TA detection in children with unexplained fevers is a promising strategy, potentially reducing the interval between disease onset and diagnosis, which in our study showed a trend toward association with refractory and relapsing TA (*p* = 0.067), although this did not reach statistical significance.

By the end of our observation period, the initial treatment regimen was effective in only 42.9% of cases (18 out of 42 patients), which was notably lower than the 67% efficacy reported in Aeschlimann et al.’s [19] study involving pediatric patients. Consequently, 24 children required intensified therapy due to the ineffectiveness of standard TA treatment (Group 2).

To identify potential indicators of TA relapse in children undergoing first-line therapy, we conducted a comparative analysis of disease onset characteristics between patients in Groups 1 and 2. Beyond the duration of disease prior to diagnosis, we observed statistically significant differences in the length of observation at our clinic; patients in Group 1 were monitored for a significantly shorter period than those in Group 2 (*p* < 0.001). This observation was partly age-related, as patients aged 18 and older transitioned to adult clinics, limiting our ability to track their long-term treatment outcomes. The shorter observation period for Group 1 patients suggests the possibility of underreported TA relapse cases due to temporal limitations in the monitoring.

When examining the onset symptoms of TA in Group 2 patients, limb claudication was found to be statistically significantly more prevalent (*p* = 0.047). Symptoms indicative of critical ischemia suggest a prolonged disease course with marked vascular wall remodeling and compromised blood supply to target organs. The distribution of vascular lesions in Group 2 patients more frequently involved the inflammatory process in the abdominal aorta and its branches.

In 91.7% of Group 2 patients, disease recurrence was confirmed through progressive vascular lesions, as verified by objective imaging. Laboratory markers of disease activity were present in 83.3% of Group 2 patients, while subjective symptoms such as malaise, headache, or vascular pain were less commonly reported.

Additionally, Group 2 exhibited higher median values for the duration of the disease prior to diagnosis, although this difference did not reach statistical significance (*p* = 0.067), suggesting a trend towards a longer diagnostic delay, particularly in cases with complex presentations. Early screening strategies should be prioritized to improve disease detection. Notably, limb claudication (*p* = 0.047) and severe ischemic symptoms were more frequent in the relapse group, suggesting a longer disease course and more excellent vascular remodeling. Patients with early signs of limb ischemia should, therefore, receive closer monitoring to adjust treatment plans promptly.

A primary objective of our research was to identify predictors of TA relapse. Our analysis indicates that an ITAS.A score exceeding 12 served as a significant predictor of relapse in our patient cohort. Further exploration revealed that combinations of five to ten parameters, including platelet levels, time to diagnosis, initial GC dosage, and age at diagnosis, demonstrated reasonable specificity and sensitivity for prognostic modeling. Further studies are warranted to identify additional or more specific factors influencing TA relapse.

The relapse-free survival rate during the 18-month observation period was merely 50%. In contrast, Goel et al. [7] reported a 66% five-year recurrence-free survival rate and a 52% ten-year survival rate among adult TA patients. Moreover, the median time to diagnosis in their study (24 months) was longer than in our cohort (11 months). In a study by Fan et al. [27], similar results were obtained. During the study, it was found that the 5-year survival rate for children without vascular complications was 45.9% and the relapse-free survival rate was 62.3%. A study by Commarmond et al. [28] found 5- and 10-year relapse-free survival rates to be 58.6% and 47.7%.

The stark discrepancy in relapse-free survival rates between our study and those of Goel et al. and Aeschlimann et al. may be attributed to several factors. First, as a federal center, we cater to children from across the nation. Many patients from remote regions who lack consistent access to our center’s facilities are primarily managed by local specialists and are often referred to us only when local treatment strategies prove ineffective. Second, the under-recognition of TA in children by medical practitioners frequently results in referrals to a rheumatologist at more advanced stages of the disease or without any prior diagnosis. Third, a potential genetic predisposition contributing to a more aggressive TA course in our patient cohort cannot be excluded [29]. Stojanovic et al. [30] found that the HLA-B*52 allele is significantly associated with TA onset in childhood and adolescence, a more severe clinical course, and a reduced response to primary therapy.

## 5. Limitation

Firstly, the sample size was relatively small, with only 42 patients included, excluding those with insufficient data. This may reduce the statistical power of the findings and affect their generalizability. To minimize the potential for bias due to the small number of patients, we used the pipeline described in the Methods Section to build models from randomly sampled data, followed by cross-validation and the calculation of our selection bias and the limitation of the ability to establish causal relationships between the identified risk factors and disease relapse. Additionally, the variability in the duration of the patient follow-up period could have impacted the accuracy of relapse reporting, as some patients in Group 1 had shorter observation periods, potentially leading to the underreporting of relapse cases and affecting this study’s conclusions about treatment efficacy. Furthermore, as a single-center study conducted at a specialized federal clinic, the findings may have limited applicability to other settings since the patient population may not fully represent the diverse demographic and clinical characteristics seen in the wider pediatric TA population. Unfortunately, our study did not include an evaluation of genetic markers in the observed subjects, which may have provided additional insights into the risk factors for relapse.

## 6. Conclusions

The key finding of this study is that an initial ITAS.A score above 12 is significantly associated with an increased risk of relapse in pediatric Takayasu’s arteritis. Further analysis identified factors such as initial platelet levels, time to diagnosis, initial glucocorticoid dosage, and age at onset as predictors of relapse. Using a random forest model, we found that combining five to ten parameters can enhance the sensitivity and specificity of relapse prediction, thereby supporting the early identification of high-risk patients and optimizing personalized treatment strategies. Future multi-center, prospective studies with larger sample sizes and genetic assessments are recommended to validate these findings and enhance their applicability.

## Figures and Tables

**Table 1:** Criteria for inclusion and exclusion.

Criteria of Inclusion	Criteria of Exclusion
Ages from 2 to 18 years old	Other diseases of the aorta and its branches
A diagnosis of TA was established under the criteria EULAR/PRINTO/PRES 2010 [10]	Social or other reasons that may prevent regular medical examinations
	Inability to receive prescribed treatment (including parental refusal to provide prescribed therapy)
	Tuberculosis
	Pregnancy or breastfeeding

**Table 2:** Generalized demographic indicators of patients in both groups. Group 1 (n = 18): no signs of relapse with first-line treatment with non-biologic DMARDs at the end of the follow-up period. Group 2 (n = 24): signs of relapse during first-line therapy with non-biologic DMARDs.

Index	Group 1 (n = 18)	Group 2 (n = 24)	*p* Value
M/F ratio	1:5	1:3.8	>0.999
Age at onset, years	11 (IQR: 10; 15)	11.5 (IQR: 8.5; 14)	0.504
Duration of TA before diagnosis, months	10 (IQR: 2; 13.75)	12.5 (IQR: 7; 24)	0.067
Duration of TA, months	30 (IQR: 22.5; 70.5)	57.5 (IQR: 46.75; 79)	0.040
Duration of observation, months	17 (IQR: 5.25; 22)	35 (IQR: 26.25; 49.25)	<0.001

**Table 3:** The frequency of clinical manifestations in children with TA, n = 45.

Clinical Manifestations	Total		Group 1		Group 2		*p*
	n = 45	%	n = 18	%	n = 24	%	
Malaise	41	91.1	17	94.4	21	87.5	0.623
Fever	31	68.9	14	77.8	14	58.3	0.186
Headache	24	53.3	9	50	12	50	0.789
Vascular pain	19	42.2	7	38.9	10	41.7	0.856
Weakening and/or absence of pulse	15	33.3	6	33.3	9	37.5	>0.999
Limb claudication	14	31.1	3	16.7	11	45.8	0.047
Weight loss	14	31.1	7	38.9	5	20.8	0.335
Arthralgia	11	24.4	3	16.7	6	25	>0.999
Abdominal pain	9	20	1	5.6	8	33.3	0.109
Limb numbness	9	20	4	22.2	5	20.8	>0.999
Carotidynia	8	17.8	3	16.7	5	20.8	>0.999
Myalgias	6	13.3	2	11.1	3	12.5	0.676
Dizziness	3	6.7	2	11.1	1	4.2	0.567
Lymphadenopathy	2	4.4	1	5.6	1	4.2	>0.999
Syncope	2	4.4	0	0	2	8.3	0.131
Arthritis	2	4.4	1	5.6	1	4.2	>0.999
Violation of visual acuity	2	4.4	1	5.6	1	4.2	>0.999
Hearing loss	1	2.2	1	5.6	0	0	0.429
Nausea	1	2.2	1	5.6	0	0	0.890
Nose bleeding	1	2.2	0	0	1	4.2	>0.999

**Table 4:** The frequency of vascular lesions in TA in pediatric patients at diagnosis, n = 45.

Affected Vessels	Total		Group 1		Group 2		*p*
	n = 45	%	n = 18	%	n = 24	%	
Left carotid arteries	30	66.7	13	72.2	14	58.3	0.353
Abdominal aorta	23	51.1	8	44.4	14	58.3	0.372
Left subclavian artery	23	51.1	8	44.4	13	54.2	0.533
Right carotid arteries	21	46.7	7	38.9	12	50	0.474
Celiac trunk	17	37.8	6	33.3	10	41.7	0.582
Descending thoracic aorta	16	35.6	5	27.8	11	45.8	0.233
Brachiocephalic trunk	13	28.9	5	27.8	5	20.8	0.720
Superior mesenteric artery	12	26.7	2	11.1	9	37.5	0.080
Left vertebral artery	11	24.4	4	22.2	6	25	>0.999
Left renal artery	10	22.2	3	16.7	7	29.2	0.473
Ascending aorta	8	17.8	3	16.7	5	20.8	>0.999
Right renal artery	7	15.6	2	11.1	5	20.8	0.679
Right subclavian artery	7	15.6	2	11.1	4	16.7	0.685
Aortic arch	6	13.3	2	11.1	3	12.5	>0.999
Right vertebral artery	3	6.7	2	11.1	1	4.2	0.567
Iliac arteries	3	6.7	0	0	2	8.3	0.498
Axillary arteries	1	2.2	0	0	1	4.2	>0.999
Femoral arteries	1	2.2	0	0	1	4.2	>0.999
Splenic artery	1	2.2	0	0	1	4.2	>0.999

**Table 5:** Median time to relapse in corresponding subgroups.

Subgroup	Median Time to Relapse and 95% CI
ITAS.A < 12	60 [19; -] months
ITAS.A >= 12	12 [9; 22] months

**Table 6:** Predictors of relapse with median importance.

N	Factor	Coeff
1	ITAS.A at diagnosis	0.033865
2	PLT at diagnosis	0.02988
3	Time to diagnosis	0.02988
4	GC dose at diagnosis	0.02988
5	Age at onset	0.015936
6	WBC at diagnosis	0.013944
7	Fever at onset	0.00996
8	Bruits at diagnosis	0.00996
9	CRP at diagnosis	0.007968
10	Arterial hypertension at diagnosis	0.007968
11	ESR at diagnosis	0.005976
12	HGB at diagnosis	0.005976
13	Asymmetry of BP at diagnosis	0.003984
14	Abdominal pain at onset	0.003984
15	Vascular pain at onset	0.001992
16	Claudication at onset	0.001992
17	Carotidynia	0.001992
18	Weight loss	0
19	Dizziness	0
20	Syncope	0

**Table 7:** The value of the concordance index and average ROC-AUC depending on the number of predictors in the models.

Number of Predictors	Median ROC-AUC, 95% CI	Mean C-Index
5	1 [0.892; 1]	0.884
10	1 [0.853; 1]	0.894
15	1 [0.882; 1]	0.906

## Data Availability

The datasets used and/or analyzed during the current study are available from the corresponding author upon reasonable request. The data are not publicly available due to being a part of an ongoing study.
